# O-terminated interface for thickness-insensitive transport properties of aluminum oxide Josephson junctions

**DOI:** 10.1038/s41598-022-16126-1

**Published:** 2022-07-12

**Authors:** Zheng Shan, Xuelian Gou, Huihui Sun, Shuya Wang, Jiandong Shang, Lin Han

**Affiliations:** 1 State Key Laboratory of Mathematical Engineering and Advanced Computing, Zhengzhou, 450001 Henan China; 2 National Supercomputing Center in Zhengzhou, Zhengzhou University, Zhengzhou, 450001 Henan China; 3 Songshan Laboratory, Zhengzhou, 450046 Henan China; 4 School of Computer and Artificial Intelligence, Zhengzhou University, Zhengzhou, 450001 Henan China

**Keywords:** Chemical physics, Condensed-matter physics, Quantum physics

## Abstract

Alumina Josephson junction has demonstrated a tremendous potential to realize superconducting qubits. Further progress towards scalable superconducting qubits urgently needs to be guided by novel analysis mechanisms or methods to reduce the thickness sensitivity of the junction critical current to the tunnel barrier. Here, it is first revealed that the termination mode of AlO_x_ interface plays a crucial role in the uniformity of critical current, and we demonstrate that the O-terminated interface has the lowest resistance sensitivity to thickness. More impressively, we developed atomically structured three-dimensional models and calculated their transport properties using a combination of quantum ballistic transport theory with first-principles DFT and NEGF to examine the effects of the Al_2_O_3_ termination mode and thickness variations. This work clarifies that O-terminated interface can effectively improve the resistance uniformity of Josephson junction, offering useful guidance for increasing the yield of fixed-frequency multi-qubit quantum chips which require tight control on qubit frequency.

## Introduction

Josephson junctions are key components for building a superconducting-circuit based quantum computer, which garners immense interests from both academia and industry. Functioning as a non-linear inductor, the Josephson junction allows two states in an anharmonic potential to be selected as the discrete computational basis for a qubit^1^. The junctions are usually fabricated from Al/AlO_x_/Al trilayer structures, which have proven to be among the best developed Josephson junctions for quantum computers^2^. The AlO_x_ tunnel barrier must be ultrathin (~ 1 nm) in order to maintain phase coherence across the superconductors and facilitate efficient quantum transport between the superconductors^3^. As the scale of superconducting quantum circuits grows, fixed-frequency qubits are attractive for their long coherence and low-cost calibration. The principal challenge for scaling fixed-frequency qubits is frequency collisions. Precise control of qubit frequencies demands that the Josephson junction conductance must be controlled with high precision^4^. However, current fabrication techniques limit the ability of predictive control over the transport properties of Josephson junctions^5^. This is mainly because structural defects in the contact interface between aluminum and alumina^6^, the roughness of the lower aluminum electrode^7^, macroscopic surface roughness^8^ and the variations of the alumina thickness^9,10^ can affect the efficiency of quantum transport of aluminum oxide Josephson junctions. Better understanding of the microscopic details related to these inhomogeneities and how they may be controlled is a present focus.

Current efforts towards this goal can be loosely sorted into two main categories. One focuses on empirical fabrication improvements in the convenience of advanced characterization devices. Low substrate temperatures and high deposition rates could improve the homogeneity of the lower Al layer^11^. Ultrasonically assisted development, uniform ashing, and dynamic oxidation were found to be helpful for improving absolute level of resistance variations^12^. The oxidation time had a greater effect on the inhomogeneity of the potential barrier thickness distribution than the oxygen pressure^9^, and the thickness variation of the alumina layer was partly attributed to grain-boundary grooves in the lower aluminum electrode layer^13,14^. By applying transmission electron microscopy in combination with electron energy loss spectroscopy, structural and nanochemical properties of differently fabricated AlO_x_ layers were analyzed and correlated with fabrication parameters^15^. These studies provide an understanding of junction growth mechanisms and fabrication control. The analysis of the junction at the macroscopic level facilitates the adjustment of the process parameters to improve its homogeneity, but the absence of theoretical mechanisms at this level is not conducive to the improvement of the fabrication process.

Another approach is computational modeling, which is low cost and high-throughput over the former experimental method. Simmons model was used to describe the conductance of Al/AlO_x_/Al junction, which did not require excessive computational resources, but the presence of empirical parameters inevitably led to errors and inaccuracies in the calculation results^16^. Jung et al.^17^ calculated parameters from first-principles to fit experimental data. Koberidze et al.^18^ investigated how six different Al/Al_2_O_3_ interfaces affected transport properties through the potential barrier at the microscopic level by constructing an atomic model of a heterojunction. Koberidze et al.^19^ showed that alumina surface irregularities and interfacial and internal relaxation affected the width and height of the barrier layer, thus altering the uniformity of the oxide layer and having a serious impact on the device performance. The transport properties of alumina tunnel junctions were simulated with non-equilibrium Green’s functions (NEGF) by Cyster et al.^20^, which indicated an exponential dependence of junction resistance on oxide density. DuBois et al.^21^ presented a methodology for constructing atomic-scale computational models of Josephson junctions using a combination of molecular mechanics, empirical and ab initio methods. Diešková et al.^22^ performed a computational study of possible local geometric structures of interfaces at the ab-initio DFT/GGA level of approximation to complement recent experimental data on ultra-thin AlO_x_-based interfaces. Even small atomic rearrangements on the AlO_x_-based interfaces played a significant rule in transport properties of Josephson junctions. Despite numerous efforts on the better control of the junction homogeneity both from experimental and computational perspectives, an adequate alumina interface is still absent. In comparison with the materials-centric history of classical digital computing, quantum systems have yet to discover their silicon and silicon oxide-the perfect interface between silicon and silicon oxide is the main reason for the silicon era.

Here, we reveal for the first time that the termination mode of the Al_2_O_3_ interface plays a crucial role in the transport properties of the junction, and demonstrate that the conductance of the O-terminated mode has the lowest sensitivity to thickness. We developed atomically structured three-dimensional α-Al_2_O_3_ models and calculated their transport properties using a combination of quantum ballistic transport theory with first-principles DFT and NEGF to examine the effects of the Al_2_O_3_ termination mode and thickness variations. Furthermore, the termination mode had a more significant effect on transport properties than the thickness of similar oxide layers and the O-terminated interface can effectively improve the resistance uniformity of Josephson junction. Here, we emphasize the impact of interfacial termination mode on the transport properties of Al/AlO_x_/Al Josephson junctions using three-dimensional full-device models, providing valuable insights into solving the frequency deviations problem.

## Results

### Designing atomic-scale models of Josephson junctions with different termination modes

**Figure 1:**
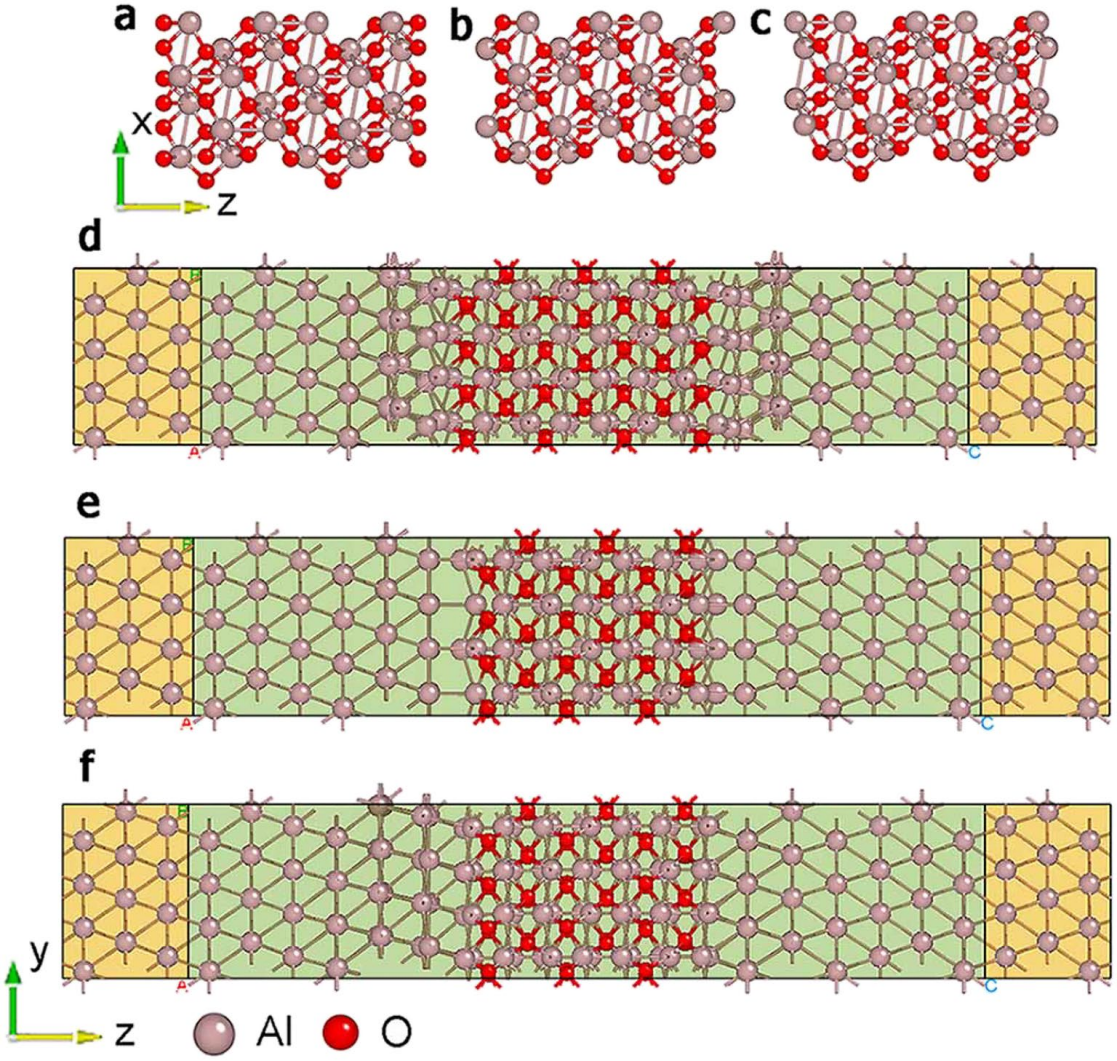
End views and side views of Josephson junction models with different termination modes. (**a**) End view of O-terminated model. (**b**) End view of Al-terminated model. (**c**) End view of 2Al-terminated model. (**d**) Side view of O-terminated model. (**e**) Side view of Al-terminated model. (**f**) Side view of 2Al-terminated model. Red spheres denote O atoms and pink spheres denote Al atoms.

The atomic-scale models of Josephson junctions are based on the first-principle methods, as implemented in the Nanodcal software^23^. Previous studies have shown that matched Al(111)/α-Al_2_O_3_(0001) interfaces are the most stable structures^24,25^. Therefore, we used Al(111)/α-Al_2_O_3_(0001)/Al(111) models shown in Fig. 1, where the x- and y- directions were periodic and electrical transport was along the z-direction. The structures had left and right electrodes and a central scattering region. The electrodes extended to $z=\pm \infty$, where bias voltages were applied and electric currents were collected. The periodic structure had a semi-infinite length. Buffer layers in the boundary areas of the scattering region shielded the electrodes from scattering effects^23^, and the buffer layer material was consistent with that of the electrodes. Three different termination modes were included for the Al_2_O_3_(0001) surface^28,31,32^: O-terminated mode via one layer of O atoms, Al-terminated mode by one layer of Al atoms, and 2Al-terminated mode via two layers of Al atoms.

We used the 4.050 Å lattice constant of bulk Al for *a* = *b* = *c*, in good agreement with experimental and calculated data^26,27^. The lattice constants of α-Al_2_O_3_ were *a* = 4.759, *b* = 8.243, and *c* = 12.991 Å, in good agreement with previous calculations^25–30^.

We also investigated the effects of various termination modes on junction transport properties for various Al_2_O_3_ thicknesses. We fixed the left side of Al_2_O_3_ as O-, Al-, and 2Al-terminated modes, and increased tunnel barrier thicknesses on the right side layer by layer with O, Al, 2Al-terminated modes respectively. 12 models with different thicknesses were obtained with the same termination mode on the left side of Al_2_O_3_, and 36 models with different thicknesses were calculated in total.

### Zero-bias conductance for different termination models

**Figure 2:**
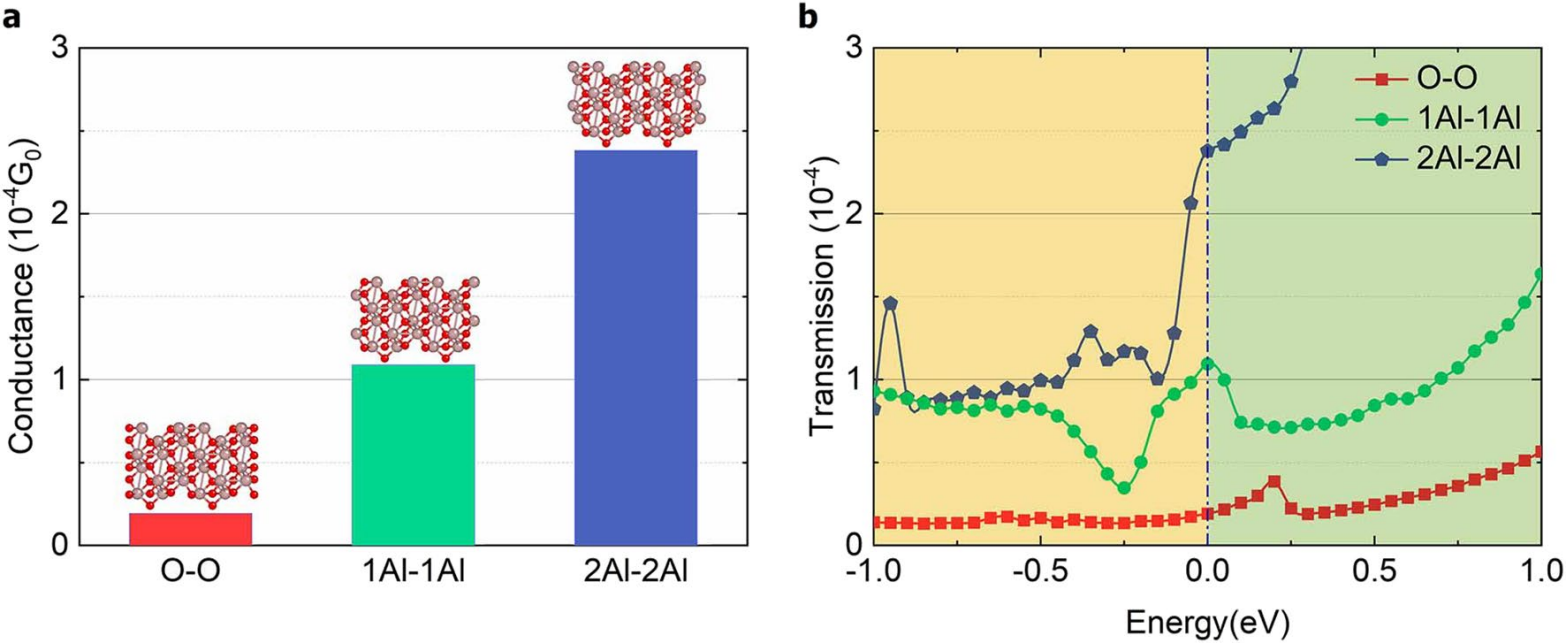
Conductance and transmission spectra of junction models with different termination modes at zero-bias voltage. (**a**) Zero-bias conductance of the O-terminated (O), Al-terminated (1Al), and 2Al-terminated models (2Al). (**b**) Transmission spectra at zero-bias voltage for the three models, where the red, green, and blue lines indicated O-, Al-, and 2Al-terminated on both sides of the Al_2_O_3_ models respectively. The transmission coefficients at zero-bias voltage were consistent with the zero-bias conductance.

To qualitatively characterize the conductance differences between the device models with three different Al_2_O_3_ termination modes, we first calculated the conductance without applying any bias voltage, i.e., zero-bias voltage. The zero-bias conductance for models with different interfacial termination modes is shown in Fig. 2a. Figure 2a shows that the conductance of the three models differ significantly as the oxide layer is extremely thin, which verifies that the junction conductance is very sensitive to interfacial effects as suggested by Müller et al^33^. The conductance of O-terminated model is an order of magnitude lower than the other two models, and the 2Al-terminated model has the largest conductance. These values are in good agreement with the transmission coefficients near the Fermi level in the equilibrium state, as shown in Fig. 2b, where the transmission is the greatest for the 2Al-terminated model and lowest for the O-terminated model. This may be because the Al- and 2Al-terminated models form metallic channels, enabling electrons to pass through. In contrast, the O-terminated junction model has the smallest transmission coefficient at the Fermi energy (0 eV), resulting in the smallest conductance.

### *I*−*V* curves for models with different termination modes

**Figure 3:**
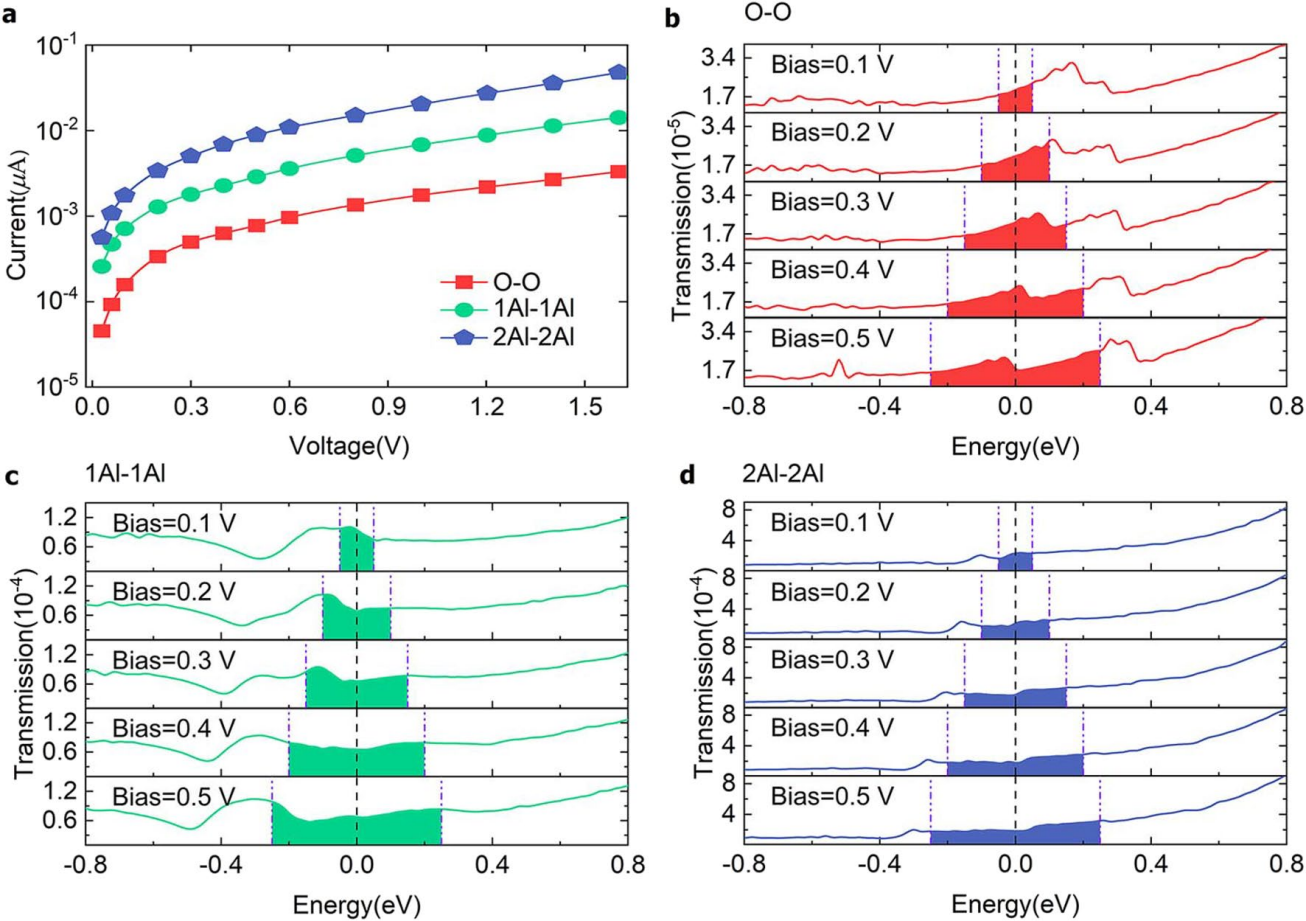
Current-voltage curves and transmission spectra of models with different termination modes at different bias voltages. (**a**) Current-voltage (*I*−*V*) curves of three calculated Al_2_O_3_ termination mode device models. The red, green, and blue lines denote the O-terminated (O), Al-terminated(1Al), and 2Al-terminated (2Al) oxide layer models respectively. (**b**) Transmission spectra of O-terminated model. (**c**) Transmission spectra of Al-terminated model. (**d**) Transmission spectra of 2Al-terminated model.

To explore the relationship between the current changes as the applied bias voltage increases, we calculated the *I-V* curves in the range of 0.03–1.6 V using Landauer formalism, as shown in Fig. 3a. The currents of all three models exhibits linearly increasing ohmic behavior when the voltage is close to 0 V, and an exponential increase at high voltages. This is consistent with experimental measurements^34^ and previous calculations for thin-film tunneling^35^. Transmission spectra for the O-, Al-, and 2Al-terminated models at different voltages are shown in Fig. 3b–d, where the purple dashed lines at the left and right ends correspond to the bias window. The energy levels of Al_2_O_3_ and Al are different at different biases. When the bias window is small, the transmission spectrum is flat and the current increase almost linearly. The transmission coefficients increase significantly when the bias window is large, and new peaks appear because the number of channels that participate in electron conduction is not the same under different biases and the transmission of each channel is not necessarily the same, which indicates that the current increases non-linearly with increasing biases, in agreement with the *I*−*V* curve in Fig. 3a. The transmission of 2Al-terminated model is relatively large, which corresponds to the large current obtained from the Landauer formalism.

### Forward and reverse *I*−*V* curves for different termination models

**Figure 4:**
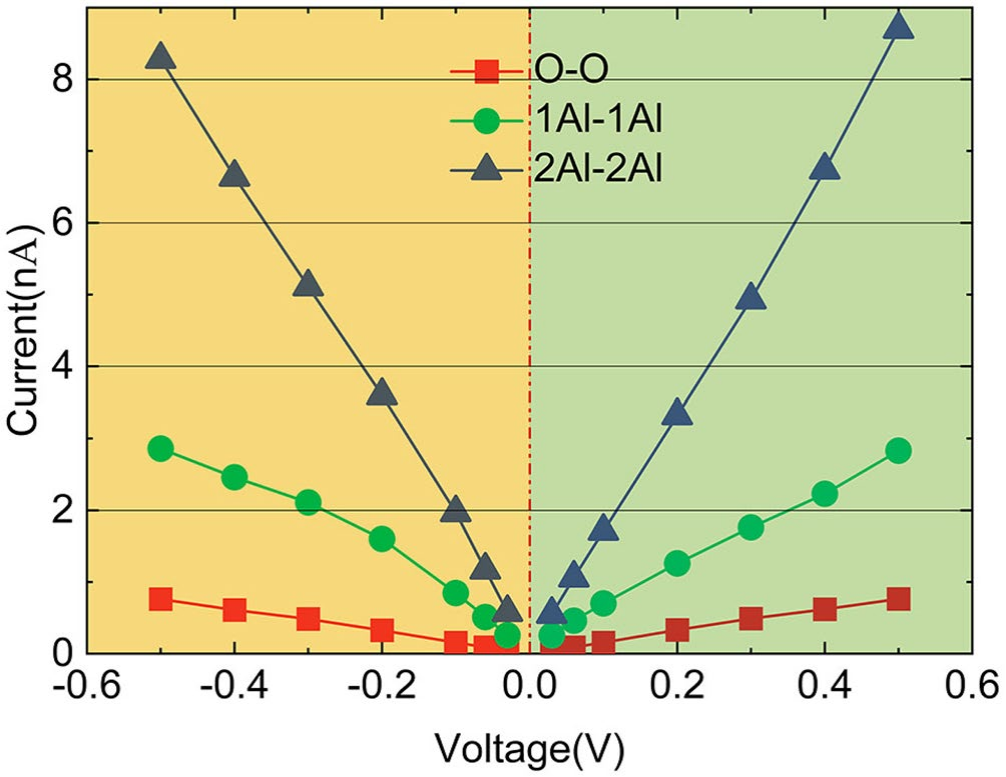
*I-V* curves for the three different oxide termination models for different voltage polarities. The red, green, and blue lines denote the O-terminated (O), Al-terminated(1Al), and 2Al-terminated (2Al) oxide layer models on both sides of Al_2_O_3_, respectively.

To clarify whether the asymmetry of the internal atomic structure has an important effect on the transport properties of the Josephson junctions, we calculated the forward and reverse currents of the models. Voltages in opposite polarities were applied to each of the three device models to determine differences between forward and reverse currents. The forward and reverse *I*−*V* curves for the O-, Al-, and 2Al-terminated models are shown in Fig. 4. Small differences in the forward and reverse currents are qualitatively consistent with previous measurements^17^. In the transmission spectra shown in Fig. 3b–d, the chemical potential of the whole system is different when the applied voltage is opposite in polarity. This corresponds to the integration of the spectra over a certain range on the left and right sides of the Fermi-energy level. The spectra are not perfectly symmetrical on both sides of that level because of small differences in the interface structures at the two ends of Al_2_O_3_. Therefore, the current obtained by integrating the spectrum over a certain range through Eq. 8 differs when various voltage polarities are applied. However, these small differences have no effect on the qualitative analysis of the junctions. O-terminated model has the smallest difference, while Al-terminated model has the largest difference, indicating that different termination modes affect the responses of the different models to various voltage polarities.

### Conductance of junction models with various thicknesses

**Figure 5:**
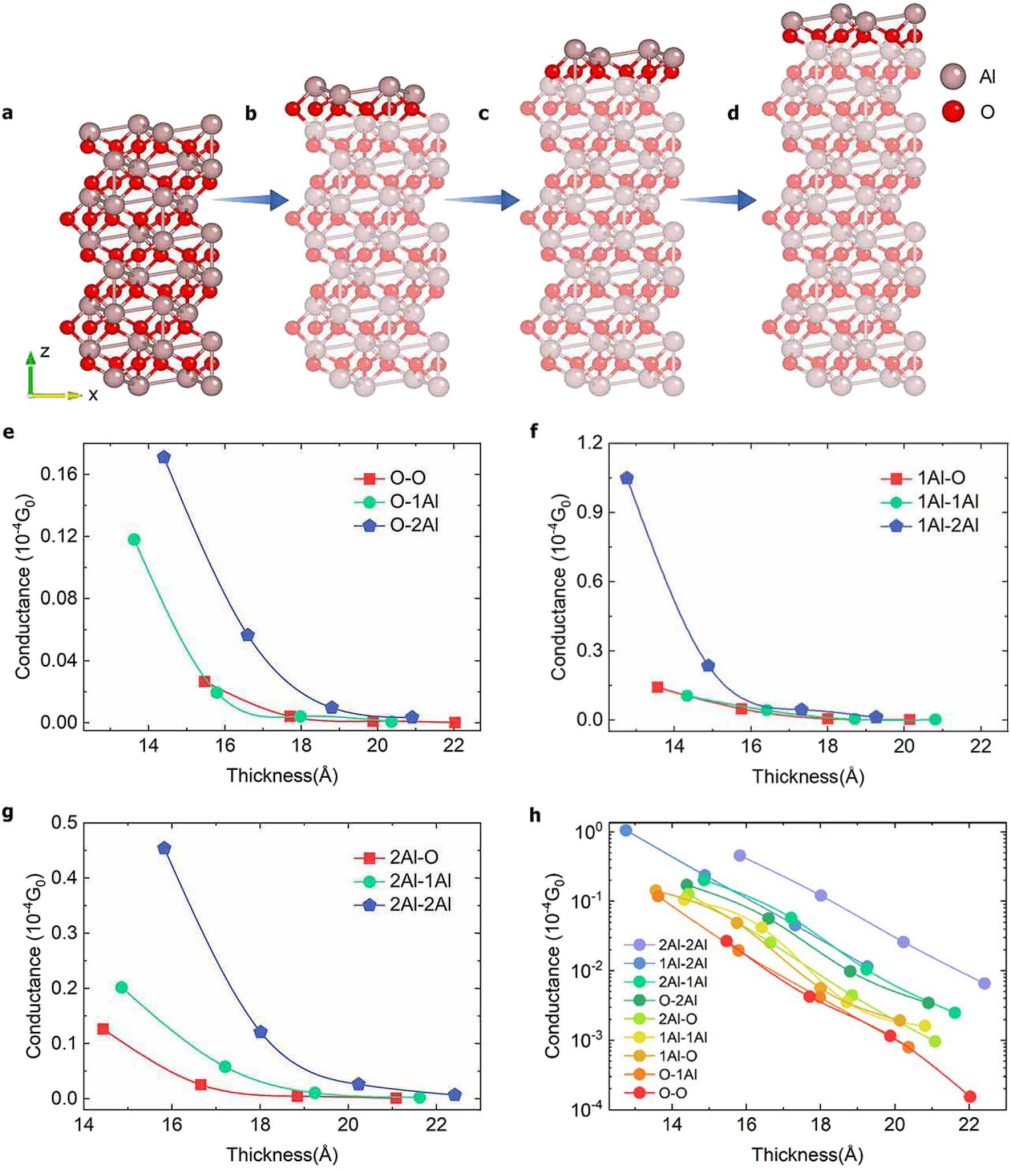
Atomic structure of Al_2_O_3_ and zero-bias conductance for different thickness models. (**a**–**d**) Portions of four different oxide layer thicknesses for the 2Al-2Al model. Zero-bias conductance versus oxide thickness for O (**e**), 1Al (**f**), and 2Al (**g**), on the left side, where the red, green, and blue lines denote O-, Al-, and 2Al- termination on both sides of Al_2_O_3_ respectively. (**h**) Zero-bias conductance for all termination models for different thicknesses.

To qualitatively characterize the differences in conductance of various Al_2_O_3_ termination modes with different oxide layer thicknesses, we calculated the zero-bias conductance for each model. We fixed the left side of Al_2_O_3_ as O, 1Al, and 2Al termination modes, and increased the O-, Al- and 2Al-termination oxide layer thicknesses on its right side layer-by-layer to obtain a total of 36 three-dimensional models with different thicknesses. The termination modes on both sides of the Al_2_O_3_ are not the same, including 9 different termination modes in total, that is, 2Al-2Al, 1Al-2Al, 2Al-1Al, O-2Al, 2Al-O, 1Al-1Al, 1Al-O, O-1Al and O-O terminated at the left and right sides of Al_2_O_3_. The structure of junction oxide layers terminated with 2Al at both ends for different thicknesses are shown in Fig. 5a–d. We calculated the zero-bias conductance of the models having the same termination on the right side, while having the O-, Al- and 2Al-terminated models on the left side respectively, as shown in Fig. 5e–g.

Figure 5e–g shows that the zero-bias conductance decays exponentially with the thicknesses of the potential barrier layer increasing, which is in good agreement with the theory and previous reports^16^. The conductance with 2Al on the right side is larger because of metal channels and thus increased electron tunneling probabilities. For comparison, the zero-bias conductance for different thicknesses for all the 9 models are plotted in Fig. 5h. The best conductance of the device models is achieved when both ends of the oxide layers are structured with 2Al termination. The conductance is worst when both ends were O termination, and the conductance of all the models decreases exponentially because the probabilities of carrier tunneling decrease sharply. The termination mode plays a dominant role in determining the conductance of different junction models with similar thicknesses. For example, when the thickness of 2Al-terminated oxide layer is 22 Å, the conductance is larger than that of the O-terminated model having an oxide layer thickness of 20 Å. This may be because the oxide layer terminated with 2Al has metallic properties, while the oxide is an insulator in general. The termination mode of the alumina/Al interfaces has a significant effect on the transport properties, which suggests improvement possibilities in the fabrication process of Josephson junctions.

### Relative change rates of conductance for models with different thicknesses

**Figure 6:**
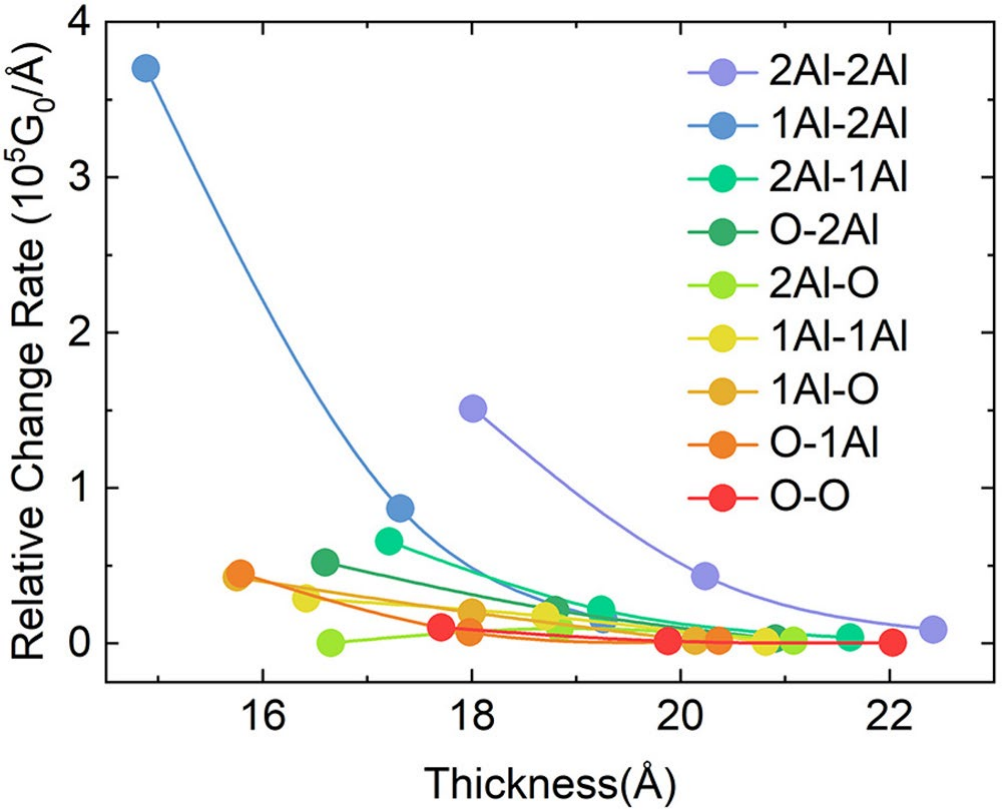
Relative change rates of conductance with increasing thicknesses for 9 termination mode models.

Small changes in the oxide layer thicknesses could significantly affect the junction conductance^37^. To evaluate this sensitivity, we calculated the relative conductance change rates against the barrier thickness for different alumina termination modes at zero bias. We defined the relative conductance change rate as $(G_n-G_{n+1})/d$ Å, where $G_n$ is the conductance of a junction with a Al_2_O_3_ barrier of *n* layers, and *d* is fixed at 22 Å, at which the conductance is almost invariant with the increasing thickness. As shown in Fig. 6, for 9 different oxide termination modes, the change rates for each mode decreases with the increasing thickness. Noticeably, the O–O model shows a lowest change rate, while the 2Al model has the highest one. This indicates that the O–O model is more insensitive to thickness, as compared to other models.

## Discussion

The structural and nanochemical properties of thin AlO_x_ layers are decisive for the transport properties of Josephson junctions, which are crucial for qubit frequencies and the scaling of superconducting qubits. In this paper, we characterized three different Al_2_O_3_ interface termination modes, and calculated their zero-bias conductance and the *I-V* curves accordingly. In addition, the effects of termination modes on junction transport properties for different thicknesses were also considered. The results indicate that the interfacial termination mode of Al_2_O_3_ in the Al/Al_2_O_3_/Al system greatly affects the electronic transmission and the zero-bias conductance. The 2Al-terminated models have the highest conductance, while O-terminated models have the lowest conductance. The conductance is exponentially dependent on the oxide thickness regardless of the termination mode of the system. In a comparison between the thickness of Al_2_O_3_ and the termination mode, the termination mode has a greater effect on the transport properties. In addition, there is a significant difference in the sensitivity of the different termination modes to the Al_2_O_3_ thicknesses, where the O-termination model has the lowest sensitivity. In summary, by combining first-principle density functional theory and non-equilibrium Green’s function theory in the calculations, we provided a clearer understanding of the important effects of interfacial termination modes and Al_2_O_3_ thicknesses on transport properties in the Al/Al_2_O_3_/Al system.

**Figure 7:**
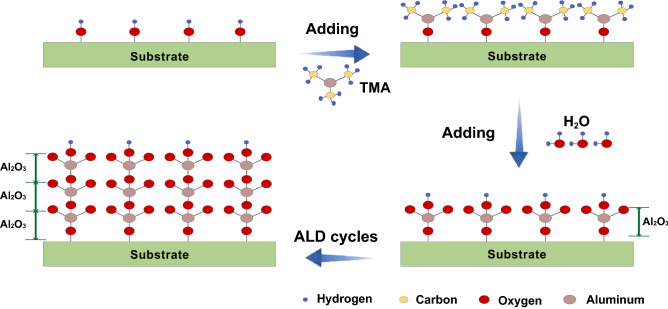
Schematic diagram of ALD process for fabricating Al_2_O_3_ film^38^.

To enhance the impact of our conclusions, we discuss experimental possibilities to control the termination of Al_2_O_3_ barriers. Two mainstream fabrication techniques for oxide layers of junctions are thermal oxidation and Atomic Layer Deposition (ALD). For the thermal oxidation process, there are barely no previous studies about precise control of termination modes not even alumina thicknesses. For the ALD process, the thickness of the oxide layer can be precisely controlled and the oxide layer is O-terminated in theory according to the chemical reaction formula^37^ as follows.

1 $${\text{AlOH}}^{*} + {\text{Al}}\left( {{\text{CH}}_{3} } \right)_{3} \to {\text{AlOAl}}\left( {{\text{CH}}_{3} } \right)_{2}^{*} + {\text{CH}}_{4}$$

2 $${\text{AlCH}}_{3}^{*} + {\text{H}}_{2} {\text{O}} \to {\text{AlOH}}^{*} + {\text{CH}}_{4}$$

where asterisks denote surface species. The process of alumina fabrication process using ALD is shown in Fig. 7. Firstly, an atomic layer terminated by hydroxyl groups is formed on the surface of the substrate. Then trimethylaluminum (TMA) is introduced, which reacts with the surface hydroxyl groups (OH^−^) to deposit a layer of $Al(CH_3)_{2}$ on the surface. Then, inert gas is introduced and water is hydroxylated on the surface reactant again, thus forming a complete cycle. Repeated this process can obtain a continuous and uniform high-quality Al_2_O_3_ film.

Based on the chemical reaction formula, it is theoretically possible to fabricate Al_2_O_3_ films in the O-terminated mode by ALD, so that Josephson junctions with lower thickness sensitivity can be obtained, thus improving the resistance uniformity of Josephson junctions. However, the current experimentally fabricated Josephson junctions using ALD techniques do not have ideally O-terminated oxide layers due to inevitably formed oxide layers in between the Al films forming process and ALD in addition to other fabrication limits. Although precise control of the alumina termination modes cannot be achieved using current fabrication techniques, the schematic diagram of ALD process for fabricating oxide layers proves that it is theoretically feasible to control the termination mode to be O-terminated which is favorable for precise control of qubit frequencies. We expect that in the near future, fabrication process might be improved towards controlling the termination modes of the oxide layers.

## Methods

### The relation between qubit frequencies and transport properties of Josephson junctions

Qubit frequency follows $\omega _{01}=$ $(\sqrt{8E_JE_C}-E_C)/\hbar$, where $E_J$ is many times greater than $E_C$ and $E_J=\frac{\hbar I_C}{2e}$. The critical current $I_C$ is set by the tunnel barrier of area ~ 200 × 200 nm and 1–3 nm thickness and is thus challenging to fabricate with precision better than a few percent. According to the Ambegaokar-Baratoff relation^39^ $I_C=\frac{\pi \Delta }{2eR_n}$ (where $\Delta$ is the superconducting gap energy), normal state resistance $R_n$ relates to $I_C$ and is readily measurable to precision better than 0.1%.

### The NEGF-DFT method

 To calculate the transmission coefficient T(E) that an electron at energy *E* scatters from the left electrode (*l*) to the right electrode (*r*), we first calculated the Green’s function:

3 $$G^{{R,A}} \left( E \right) = \left[ {ES - H - \Sigma ^{{R,A}} } \right]^{{ - 1}}$$

where $G^R$ and $G^A$ denote the retarded and advanced Green’s functions of the model respectively, S is the overlap matrix due to orbital non-orthogonality (if the orbits are orthogonal, S is the unit matrix), H is the Hamiltonian matrix of the system, $\mathrm {\Sigma }^R$ and $\mathrm {\Sigma }^A$ denote the delayed and overrunning self-energy respectively. $G^R$ can be calculated from a matrix transformation using $G^A={(G^R)}^\dag$. With the Green’s functions, the transmission coefficient was calculated with:

4 $$T(E) = tr\left[ {G^{R} (E)\Gamma _{l} (E)G^{A} (E)\Gamma _{r} (E)} \right],$$

5 $$\Gamma _{l} (E) = i\left[ {\sum\limits_{l}^{R} {(E)} - \sum\limits_{l}^{A} {(E)} } \right],$$

where $\sum _{l}^{R}{(E)}(\sum _{l}^{A}{(E)})$ is the retarded (advanced) self-energy of electrode *l*. The Brillouin zone sampling k-point was set to 20 × 20 × 1 and 100 × 100 × 1 for these calculations respectively. When the results of the two data sets were compared, the accuracy differed by only 1%, while the calculation speed was improved by a factor of twelve when the k-point was set to 20 × 20 × 1.

The conductance is given by:

6 $$G = G_{0} \int {dE\frac{{f_{l} (E) - f_{r} (E)}}{{eV_{r} - eV_{l} }}T(E)}$$

where $G_0=\frac{2e^2}{h}$ is the conductance quanta, h is Planck constant, e is the electron charge, $f_l(E)-f_r(E)$ is the difference between the Fermi distributions of the left and right electrodes, $V_r-V_l$ is the difference between the bias applied to the left and right electrodes, and T(E) is the transmission coefficient at a certain energy E. When the bias approached zero, the limit was also the conductance at equilibrium.

The conductance is related to the transmission of the carriers, where the tunneling probability of the carriers through the one-dimensional square potential barrier is given by:

7 $$D = D_{0} e^{{ - \frac{{2a}}{h}\sqrt {2m(U_{0} - E)} }}$$

where $D_0$ is a constant that is close to 1, *a* represents the barrier width (which may be different from the physical thickness of the oxide layer), and $U_0$ is the barrier height. Hence, the transmission coefficient decreases sharply if the barrier width or height is increased, while the barrier height is determined by the thickness and nature of the insulating layer. However, the one-dimensional square potential barrier does not completely describe our model because there are effects of various interface terminations. It is usually described by a trapezoidal potential barrier^18^, but the effects of barrier width on the conductivity are qualitatively consistent.

The current is given by the Landauer formalism:

8 $$I = \frac{{2e}}{h}dE\left[ {f_{l} (E) - f_{r} (E)} \right]T(E)$$

which indicates that the current is independent of how the voltage is applied, but only related to the difference noted above. The current could be considered a projection of the transmission spectrum in a certain energy range.

### Quantum ballistic transport theory

Our system is a mesoscopic system between macroscopic and microscopic, which can be obtained from experimental fabrications. The length of the middle Al_2_O_3_ part of the system is only a few nanometers, which is much smaller than the average free range of electron motion, when the electron transport process can be considered as quantum ballistic transport^40^. In the actual process, when the Al_2_O_3_ length is short enough, the system will still be affected by temperature, material defects and other factors, which may lead to diffusive transport processes, but the ballistic transport process still plays a dominant role. Therefore, our calculations on the basis of this theory for various models can achieve a qualitative analysis of the electrical transport properties of Josephson junctions.

### Protocol

 We used Nanodcal^23^ first-principle quantum-transport software based on NEGF-DFT, without empirical parameters, to calculate the electrical properties of the models and to predict current-voltage characteristics and electron transmission probabilities for various junction models.

During the self-consistent cycle, we used the Perdew-Burke-Ernzerhof functional in the generalized gradient approximation^41^. The convergence criteria for both the Hamiltonian and density matrices were set to 10^−5^ eV, and we used the linear combination of atomic orbitals (LCAO) basis set to expand the Kohn-Sham (KS) wave function, the plane wave truncation energy was set to 100 Hartree, the maximum number of steps chosen for self-consistency and the self-consistent mixing ratio were set to 200 and 0.05 respectively, and the k-point was set to 5 × 5 × 100 and 5 × 5 × 1 respectively in self-consistent calculation of electrode part and central part.

Geometric optimization was performed with a projector-augmented wave method based on DFT^42–45^. The Perdew-Burke-Ernzerhof functional in the generalized gradient approximation was used for the exchange-correlation interactions between electrons^41,46^. The cut-off energy of the plane-wave expansion was 400 eV, and the Brillouin zone was sampled with a Gamma-centered scheme, using 4 × 3 × 5 and 4 × 4 × 1 k-point sampling to optimize the Al and Al_2_O_3_ cells. The total-energy convergence criterion was at 10^−5^ eV per atom, and the maximum Hellmann-Feynman force deviation was less than 0.01 eV/Å.

Using optimized materials to build the crystal model, we varied interface contact distances and ensured that those of Al and Al_2_O_3_ at the left and right ends were the same, and calculated the variations in single-point energies with the contact distance to obtain the lowest energy point. The distance corresponding to the lowest energy was the optimum distance. The optimum interface distances for O-terminated, Al-terminated, and 2Al-terminated models were 2.9 Å, 3.1 Å, and 3 Å, respectively.

Several atom layers were optimized in the model where Al and Al_2_O_3_ were in contact. The Brillouin zone was sampled with a 4 × 3 × 1 mesh of the Gamma-centered k-point, and the remaining parameters were the same as those in the initial material optimization. Finally, models with O, Al, and 2Al terminations shown in Fig. 1 were obtained.

## Data Availability

The data that support the findings of this study are available from the corresponding author upon reasonable request.
